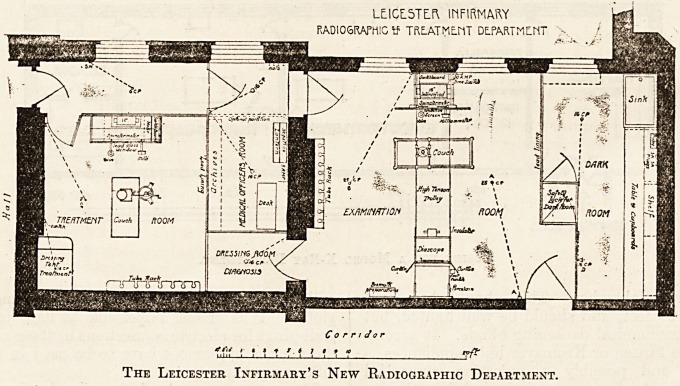# Leicester Infirmary

**Published:** 1911-06-03

**Authors:** 


### LEICESTER INFIRMARY.

### RADIOGRAPHIC AND *X*-RAY TREATMENT DEPARTMENT.

For the diagnosis of fractures, dislocations, and the detection of foreign bodies in the tissues, an *x*-ray outfit forms part of the equipment of every up-to-date General Hospital. Within recent years the use of *x*-rays in the treatment of various forms of skin diseases has rendered it necessary to have an additional outfit for this purpose.

#### Statistics.

In the Leicester Infirmary during the year 1900 229 patients were treated in the *x*-ray department. At that time the outfit consisted of an *x*-ray coil, a tube, and two storage cells for current. The number of patients treated in the *x*-ray department during the year 1909 increased to 1,122, and it was found necessary to reconstruct the whole department.

#### Description.

For completeness and convenience, the reconstructed department as shown on the plan which we reproduce compares favourably with the most up-to-date institution. The whole scheme has been well thought out, both in the interests of the patients and the nurses. The apparatus supplied is of the most modern type, and protective screens have been provided everywhere, so that the operators will not be exposed to any risk.

A room has been provided for radiography, with a dark room adjoining. The partitions are formed of fibrous plaster, faced with ordinary plaster, and, in addition, they are covered with sheet lead. A large room has also been provided for therapeutic treatment by *x*-rays, and the apparatus is of the most up-to-date pattern.

The plan does not show any waiting-room where patients can assemble, and additional dressing-room accommodation would have been a distinct advantage. The small dressing-tent in the corner of the treatment-room is not sufficient. Perhaps it may be possible to find such accommodation in close proximity to the department.

The Leicester Infirmary's New Radiographic Department.

#### Cost.

The apparatus and fittings have cost £600, and the alterations £250. The plan was prepared and the *x*-ray apparatus supplied by Mr. A. E. Dean, of London, and the alterations were carried out under the supervision of Mr. S. P. Pick, the Infirmary architect, by the Works Department of the Infirmary (Mr. Deacon). The dynamo of 100 volts is in the basement, and this and the wiring of the department were done by Messrs. Gent and Co., Ltd., Leicester. Dr. Astley V. Clarke is in charge of the department, with Mr. J. J. Hull as his assistant.

## Figures and Tables

**Figure f1:**